# Schizophrenia: can I be a normal person despite my diagnosis?

**DOI:** 10.1192/j.eurpsy.2025.2198

**Published:** 2025-08-26

**Authors:** N. Navarro, P. Martínez Gimeno, B. Rodríguez Rodríguez, M. Fernández Lozano, P. Marqués Cabeza, A. Rodríguez Campos, M. Calvo Valcárcel, M. Andreo Vidal, M. P. Pando Fernández, A. Monllor Lazarraga, G. Guerra Valera, M. Rios Vaquero, G. Lorenzo Chapatte, L. Rojas Vázquez, M. B. Arribas Simón

**Affiliations:** 1 Hospital clínico universitario de Valladolid, Valladolid, Spain

## Abstract

**Introduction:**

**Schizophrenia** is a mental disorder characterized by hallucinations, typically auditive ones, delusions, disorganized thinking and behavior, and flat or inappropiate affect. Symptoms develop gradually and usually begin during young adulthood and are never resolved completely. There is no objective diagnostic test; diagnosis is based on observed behavior, a holistic psychiatric history that includes the person’s reported experiences, and reports of others familiar with the patient.

**Objectives:**

- Explore what schizophrenia is, its main characteristics and how it differs from other mental disorders.
- Analyze how schizophrenia affects families and interpersonal relationships.

**Methods:**

Middle-aged man who resides with his adoptive parents in a town near the municipal seat, unemployed and longitudinally with few social relationships and a lover of technology. He has attended psychiatric consultations on specific occasions and irregularly without a clear diagnosis. a single admission to a city psychiatric hospital for psychotic symptoms that subsided with medication. Since then, monitoring has been erratic and he has been given medication disguised by his parents during meals. In the last two weeks he has been especially distrustful and suspicious, even with his parents, so he has barely been able to sleep and does not eat properly for fear of being poisoned. He has confined himself to his room and refuses to be evaluated by a doctor and his anguish and emotional lability are increasing. For this reason, he is taken to the emergency room, where he is evaluated and it is determined that he is going through an episode with psychotic characteristics, which is why it is necessary, given the repercussion of the condition and the patient’s lack of cooperation, to be involuntarily hospitalized for treatment and stabilization.

**Results:**

During admission, he experiences episodes of intense agitation and serious behavioral alterations that require important pharmacological adjustments. In addition, work is done on awareness of the disease and acceptance of the diagnosis. Although he is initially reluctant to take any type of intervention, he progressively accepts taking medication and understands the nature of his problem, as well as the need to continue monitoring his disorder at an outpatient level. We were also able to provide psychoeducation to the family, thus achieving commitment and support on their part as well, since at first they were reluctant that their son could suffer from schizophrenia, for fear of the stigma and rejection that this could cause.

**Conclusions:**

People with schizophrenia often face social stigmatization, which can lead to further marginalization, isolation and discrimination. This affects their emotional and mental well-being, contributing to a decrease in quality of life. Lack of adequate understanding of the disorder reinforces these myths and perpetuates discrimination.

**Disclosure of Interest:**

None Declared

